# Clinical importance of potassium intake and molecular mechanism of potassium regulation

**DOI:** 10.1007/s10157-019-01766-x

**Published:** 2019-07-17

**Authors:** Naohiro Nomura, Wakana Shoda, Shinichi Uchida

**Affiliations:** Department of Nephrology, Graduate School of Medical and Dental Sciences, Tokyo Medical and Dental University, 1-5-45 Yushima, Bunkyo, Tokyo, 113-8519 Japan

**Keywords:** Potassium, Hypertension, Sodium–chloride cotransporter

## Abstract

**Introduction:**

Potassium (K^+^) intake is intrinsically linked to blood pressure. High-K^+^ intake decreases hypertension and associated lower mortality. On the other hand, hyperkalemia causes sudden death with fatal cardiac arrhythmia and is also related to higher mortality. Renal sodium (Na^+^)–chloride (Cl^‒^) cotransporter (NCC), expressed in the distal convoluted tubule, is a key molecule in regulating urinary K^+^ excretion. K^+^ intake affects the activity of the NCC, which is related to salt-sensitive hypertension. A K^+^-restrictive diet activates NCC, and K^+^ loading suppresses NCC. Hyperpolarization caused by decreased extracellular K^+^ concentration ([K^+^]_ex_) increases K^+^ and Cl^‒^ efflux, leading to the activation of Cl^‒^-sensitive with-no-lysine (WNK) kinases and their downstream molecules, including STE20/SPS1-related proline/alanine-rich kinase (SPAK) and NCC.

**Results:**

We investigated the role of the ClC-K2 Cl^‒^ channel and its β-subunit, barttin, using barttin hypomorphic (*Bsnd*^*neo/neo*^) mice and found that these mice did not show low-K^+^-induced NCC activation and salt-sensitive hypertension. Additionally, we discovered that the suppression of NCC by K^+^ loading was regulated by another mechanism, whereby tacrolimus (a calcineurin [CaN] inhibitor) inhibited high-K^+^-induced NCC dephosphorylation and urinary K^+^ excretion. The K^+^ loading and the tacrolimus treatment did not alter the expression of WNK4 and SPAK. The depolarization induced by increased [K^+^]_ex_ activated CaN, which dephosphorylates NCC.

**Conclusions:**

We concluded that there were two independent molecular mechanisms controlling NCC activation and K^+^ excretion. This review summarizes the clinical importance of K^+^ intake and explains how NCC phosphorylation is regulated by different molecular mechanisms between the low- and the high-K^+^ condition.

**Electronic supplementary material:**

The online version of this article (10.1007/s10157-019-01766-x) contains supplementary material, which is available to authorized users.

## Introduction

Potassium (K^+^) is one of the most important electrolytes in our body, which mainly exists intracellularly. Extracellular K^+^ levels are strictly controlled within an appropriate range. Since K^+^ is essential for cell membrane potential, especially in the heart, muscles, and neurons, both hyperkalemia and hypokalemia cause heart and muscle complications, including fatal cardiac arrhythmia. The kidney is the pivotal organ that controls K^+^ balance, and urinary K^+^ excretion is regulated in the distal nephron segments. Recently, sodium (Na^+^)–chloride (Cl^‒^) cotransporter (NCC), which is expressed in the distal convoluted tubules (DCTs), has been identified as a key molecule for the regulation of urinary K^+^ excretion. The aim of this review is to summarize the clinical importance of K^+^ intake and the molecular mechanism of urinary K^+^ excretion via NCC.

## Clinical studies concerning K^+^ intake and blood pressure

It has been demonstrated in many clinical studies that K^+^ intake is inversely related to BP. In a pooled study of more than 100,000 people from 18 countries, the relationship between morning fasting urinary K^+^ excretion (a surrogate marker for K^+^ intake) and BP was analyzed [1]. The highest BP was observed in the group with Na^+^ intake of > 5 g/day and K^+^ of < 1.9 g/day, and the lowest BP occurred in the group with Na^+^ intake of < 3 g/day and K^+^ of > 2.5 g/day. Additionally, higher urinary K^+^ excretion has been associated with a lower risk of death and cardiovascular events [2, 3].

In a study of 1661 Brazilians, no apparent benefit of dietary K^+^ supplementation was evident in participants excreting < 6 g NaCl/day, and those in the highest quartile of K^+^ excretion exhibited no hypertension, supporting the idea that K^+^ intake blunts the influence of high-Na^+^ intake on BP [4]. In a meta-analysis of 33 studies where dietary K^+^ supplementation was the only intervention variable (with 2609 participants), it was concluded that K^+^ supplementation significantly reduced systolic and diastolic BP; the effects were more substantial in studies where participants consumed more Na^+^ [5]. In a recent double-blind, randomized, controlled trial, it was also demonstrated that K^+^ supplementation reduced BP [6].

Increased K^+^ intake is beneficial not only for patients with hypertension and cardiovascular disease (CVD), but also for those with chronic kidney disease (CKD). In a study on Japanese patients with type 2 diabetes, higher urinary K^+^ excretion was associated with a slower decline in renal function and CVD progression [7]. In another study evaluating 29,000 participants with vascular disease or diabetes at a high cardiovascular risk, a strong linear association between higher K^+^ intake and reduced renal outcomes over a range of intake from 1.7 to 2.7 g/day of K^+^ was observed [8]. Conversely, a study on patients with advanced CKD showed that K^+^ intake was no longer beneficial [9], suggesting that the beneficial effect of K^+^ requires the ion transporters to function normally. Additionally, a recent meta-analysis investigating the long-term observation of serum K^+^ and adverse outcomes noticeably demonstrated that higher serum K^+^ levels increased the risk of adverse outcomes, including mortality, CVD, and end-stage kidney disease [10]. Therefore, high-K^+^ intake without hyperkalemia is essential to maintain a healthy body.

## NCC is a key molecule for regulating urinary K^+^ excretion

**Fig. 1:**
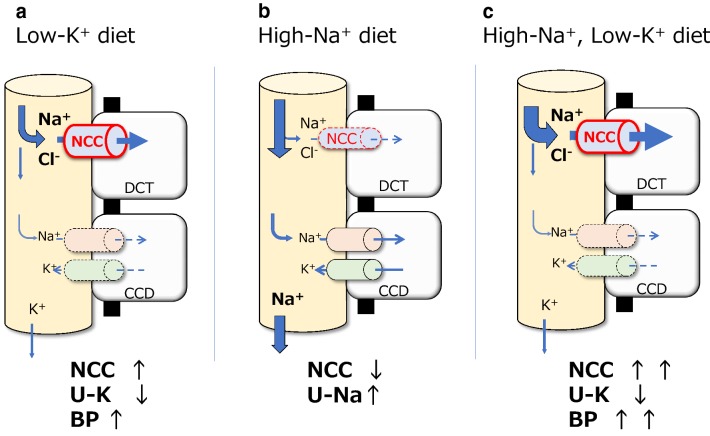
NCC regulation by different valances of Na^+^- and K^+^-containing diets. Na^+^ reabsorption via NCC in the DCT controls Na^+^ delivery to the downstream nephron segments. K^+^ is excreted by the electrical driving force generated by Na^+^ reabsorption. **a** Consuming a low-K^+^ diet activates NCC, promoting Na^+^ reabsorption, and BP elevation. **b** Consuming a high-Na^+^ diet suppresses NCC, increasing urinary Na^+^ excretion. **c** Consuming a high-Na^+^, low-K^+^ diet activates NCC despite the higher Na^+^ intake, and the greater amount of Na^+^ reabsorption causes high BP. Na^+^, sodium; K^+^, potassium; Cl^‒^, chloride; DCT, distal convoluted tubule; CCD, cortical collecting duct; NCC, sodium–chloride cotransporter; U-K^+^, urine potassium; U-Na^+^, urine sodium; BP, blood pressure

NCC has been identified as a key molecule for regulating urinary K^+^ excretion and low-K^+^-induced hypertension. This molecule is expressed in the apical membrane of DCTs and reabsorbs Na^+^ and Cl^‒^. Although NCC itself does not directly transport K^+^, the amount of NaCl reabsorption via NCC in the DCT affects the delivery of Na^+^ to the downstream nephron segments. In the downstream nephron segments, K^+^ is excreted under the effect of the electrical driving force generated by Na^+^ reabsorption via epithelial Na^+^ channels (Fig. 1). The notion that NCC is important for regulating K^+^ excretion is also supported by the fact that two genetic diseases, namely, Gitelman syndrome (caused by the loss-of-function of NCC) and pseudohypoaldosteronism type II (PHA II, caused by the gain of function of NCC), present with hypokalemia and hyperkalemia, respectively [11, 12]. In many previous studies on rodents, it has demonstrated that K^+^ intake strongly affects the total amount and phosphorylation of NCC (i.e., activity). Consuming a low-K^+^ diet increased the total amount and phosphorylation of NCC [13–18], promoting Na^+^ reabsorption and BP elevation (Fig. 1a) [13, 19]. No elevation of BP with a low-K^+^ diet was observed in NCC knockout mice and mice treated with hydrochlorothiazide (an NCC inhibitor) [13, 19], which strongly suggests that the low-K^+^-induced BP elevation was dependent on NCC.

NCC regulates not only K^+^ but also Na^+^ balance. This is evident when considering the two aforementioned NCC-related diseases. Gitelman syndrome and PHA II cause salt-losing polyuria and salt-sensitive hypertension, respectively. A high-salt diet suppresses NCC phosphorylation, and a low-salt diet promotes NCC phosphorylation (Fig. 1b) [20]. Owing to the critical importance of K^+^ balance, K^+^ regulation via the NCC mechanism occurs prior to Na^+^ regulation. A high-salt, low-K^+^ diet activates NCC despite the higher Na^+^ intake [13, 21]. Then, the increased Na^+^ reabsorption causes high BP (Fig. 1c). A high-K^+^ diet strongly suppresses low-Na^+^-induced NCC phosphorylation, with a resultant increase in urinary Na^+^ and K^+^ excretion [22]. This regulation by NCC could explain the clinical finding that a K^+^-rich diet improves salt-sensitive hypertension.

## Molecular mechanism of low-K^+^-induced NCC phosphorylation

NCC is phosphorylated and activated by STE20-related proline/alanine-rich kinase (SPAK) and oxidative-stress-responsive kinase 1 (OSR1), which, in turn, is regulated by the with-no-lysine (WNK) kinases [16, 23]. Many animal studies have shown that a low-K^+^ diet increases total and phosphorylated SPAK [13, 16, 17, 19], WNK4 [13, 19], and NCC [13-19]. Furthermore, WNK4 knockout (WNK4^‒/‒^) mice and kidney-specific SPAK/OSR1 double-knockout (SPAK^‒/‒^/KS-OSR1^‒/‒^) mice showed either no increase or only a blunted increase in phosphorylated NCC (pNCC) in response to a low-K^+^ diet, respectively [13, 16, 18]. In humans, the expression of WNK4, SPAK, and NCC was investigated using urinary exosomes [24]. It was found that the amount of WNK4, total NCC, and pNCC was negatively correlated with plasma K^+^ concentration (SPAK phosphorylation was not investigated), indicating that NCC phosphorylation with a low-K^+^ diet is dependent on the WNK4–SPAK cascade.

In recent years, it was found that WNK1 possessed a Cl^‒^-binding motif that affects WNK1 autophosphorylation (i.e., activity) [25]. These direct Cl^‒^-binding sites are situated in the catalytic sites of WNK, and their residues are conserved among WNKs. A reduction in intracellular Cl^‒^ concentration ([Cl^‒^]_in_) significantly activated WNK kinases and their downstream molecules, namely, SPAK and NCC. Mutant WNK kinases with deficient Cl^‒^-binding sites increased their autophosphorylation and then activated SPAK and NCC [15, 25, 26]. Physiologically, [Cl^‒^]_in_ is regulated by a negative basolateral membrane potential (hyperpolarization), which is the main driving force for Cl^‒^ extrusion from the cell [27]. A change in plasma K^+^ level affects the membrane potential of DCT cells, thereby altering their [Cl^‒^]_in_ [13].

Since it was proposed that Kir4.1/Kir5.1 is the predominant K^+^ channel in the basolateral membrane of DCT cells [28], this K^+^ channel was expected to contribute to Cl^‒^-sensitive WNK activation in the low-K^+^ condition. In a recent study using doxycycline-inducible kidney-specific Kir4.1 knockout mice, it was reported that the lack of Kir4.1 abolished the low-K^+^ diet-induced hyperpolarization and the increase in K^+^ conductance, resulting in decreased low-K^+^-induced NCC phosphorylation [29]. In humans, loss-of-function mutations in the gene encoding Kir4.1 cause SeSAME/EAST syndrome, characterized by an electrolyte imbalance reminiscent of Gitelman syndrome, including salt wasting, hypocalciuria, hypomagnesemia, and hypokalemic metabolic alkalosis [30]. This human genetic disease also highlights the importance of Kir4.1 for NCC activation.

**Fig. 2:**
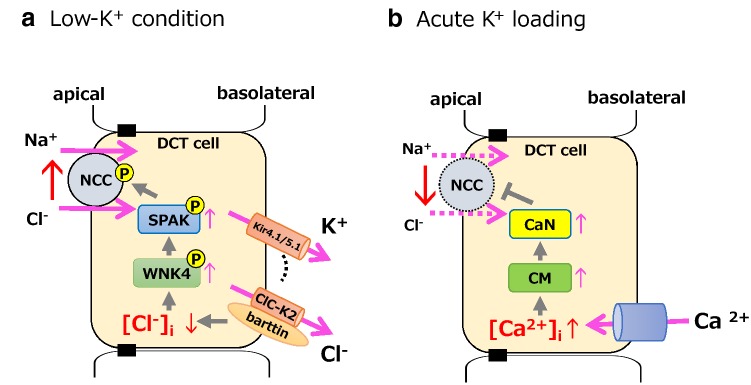
Molecular mechanism of NCC regulation by K^+^. **a** The mechanism of NCC activation under the low-K^+^ condition. The decrease in extracellular K^+^ concentration causes K^+^ efflux through Kir4.1/Kir5.1 channels. The electrical driving force generated by K^+^ extrusion causes Cl^‒^ efflux via ClC-K2/barttin. The decreased intracellular Cl^‒^ concentration activates WNK kinases. **b** The mechanism of NCC dephosphorylation by acute K^+^ loading. Depolarization caused by an increase in extracellular K^+^ concentration promotes Ca^2+^ influx, activating CM and CaN. The activated CaN then dephosphorylates NCC. Na^+^, sodium; K^+^, potassium; Cl^‒^, chloride; DCT, distal convoluted tubule; NCC, sodium–chloride cotransporter; CaN, calcineurin; CM, calmodulin

As for the Cl^‒^ channel on the basolateral membrane of the DCT, ClC-K2 (a murine ortholog of human ClC-Kb) is thought to be the predominant Cl^‒^ channel in mice, and ClC-K2 knockout mice showed a significant decrease in NCC expression [31]. In cell culture studies, the transfection of loss-of-function mutant ClC-K2 disrupted low-K^+^-induced NCC dephosphorylation [13]. We used barttin hypomorphic mice (*Bsnd*^*neo/neo*^), which are hypomorphic of a disease-causing mutant barttin (R8L), to clarify the contribution of ClC-K2 to low-K^+^-related NCC phosphorylation in vivo [21]. Since barttin is an essential β-subunit for ClC-K channels [32, 33], *Bsnd*^*neo/neo*^ mice expressed very low levels of barttin and ClC-K channels. When *Bsnd*^*neo/neo*^ mice were fed a normal diet, NCC phosphorylation was not significantly different from that of wild-type mice. Then, we fed a high-salt, low-K^+^ diet to wild-type mice and *Bsnd*^*neo/neo*^ mice. In the wild-type mice, the phosphorylation of both SPAK and NCC was significantly increased. In the *Bsnd*^*neo/neo*^ mice, however, the increase in SPAK and NCC phosphorylation was unmistakably impaired. Furthermore, the increase in BP observed in wild-type mice consuming a high-salt, low-K^+^ diet was not evident in the *Bsnd*^*neo/neo*^ mice. Thus, our study provides in vivo evidence that, in response to a low-K^+^ diet, ClC-K and barttin play vital roles in activating the WNK4–SPAK–NCC cascade and BP regulation. This low-K^+^-induced NCC phosphorylation mechanism is shown in Fig. 2a.

## **Molecular mechanism of high-K**^**+**^**-induced NCC dephosphorylation**

There have been various investigations into the effect of high K^+^ on NCC in rodent studies. Dietary intake, rapid oral gavage, and intravenous administration of KCl decreased pNCC [15, 34–36], and rapid oral gavage of KHCO_3_ also induced NCC dephosphorylation [35]. In contrast, a high-K^+^ diet with the addition of K^+^-citrate increased pNCC [14, 19] following the reduction of urinary Na^+^ excretion and BP elevation [19]. In addition to NCC phosphorylation, the effect of high K^+^ on WNK–SPAK kinase has also been controversial. One group showed a significant increase in WNK4 and phosphorylated SPAK (the accompanying anion was not described) [22]; however, other groups showed no significant change in WNK4 and phosphorylated SPAK [14, 19, 36]. To the best of our knowledge, there is no animal study demonstrating a significant reduction of SPAK and WNK kinases with a K^+^-rich diet (summarized in Online Resource 1).

To confirm the effect of an accompanying anion with K^+^ in the acute phase, we administered a K^+^ solution with different anions (KCl, K^+^-gluconate, and K^+^-citrate) to mice by oral gavage. All K^+^ solutions showed a rapid reduction of pNCC 15 min after K^+^ loading [37]. Therefore, it is necessary to consider the acute and chronic phases separately to understand the effect of high-K^+^ intake on NCC regulation, and it is possible that secondary effects of the anion accompanying K^+^ alter the response of NCC phosphorylation in chronic K^+^ loading. Next, we investigated SPAK phosphorylation after acute K^+^ loading. Although NCC was rapidly dephosphorylated after K^+^ loading, there was no significant difference in SPAK phosphorylation. This suggested that high-K^+^-induced NCC dephosphorylation was independent of the WNK–SPAK cascade, at least in the acute phase. It was concluded in a previous study that rapid NCC dephosphorylation in response to increased extracellular K^+^ was not Cl^‒^-dependent [38]. In this study, it was demonstrated that NCC phosphorylation was inversely correlated with extracellular K^+^ concentration ([K^+^]_ex_) in ex vivo kidney slices. Furthermore, it was concluded that cellular Cl^‒^ conductance and SPAK/OSR1 were involved in low-[K^+^]_ex_-induced NCC phosphorylation by observing that the removal of extracellular Cl^‒^ or the presence of 4,4′-diisothiocyano-2,2′-stilbenedisulfonic acid, a Cl^‒^ channel blocker, did not block the dephosphorylation triggered by high [K^+^]_ex_ [38].

The evidence that a high-K^+^-induced decrease in NCC phosphorylation was independent of the Cl^‒^–WNK–SPAK pathway strongly suggested the involvement of a protein phosphatase (PP). Several PPs (e.g., PP1 [39]; and calcineurin (CaN), also known as PP2B [40, 41]) have been suggested to modulate NCC dephosphorylation. PP inhibitor-1 (I-1), an endogenous inhibitor of PP1, was identified as a DCT-enriched gene product by microarray analysis of mouse DCT cells. Additionally, in an I-1 knockout mouse, in which PP1 was expected to be activated, a decrease in pNCC and significantly lower arterial BP were observed [39]. Hoorn et al. reported that tacrolimus (a CaN inhibitor) treatment significantly increased NCC phosphorylation in both mouse and human kidneys, resulting in salt-sensitive hypertension [41]. To inhibit CaN, tacrolimus must bind to a 12 kDa FK506-binding protein (FKBP12). Mice lacking FKBP12 along the nephron did not show tacrolimus-induced hypertension or increased pNCC [40].

To clarify the hypothesis that high K^+^ stimulates PPs (PP1 or CaN), leading to NCC dephosphorylation, we administered PP inhibitors to mice with a rapid oral K^+^ load [37]. Although tautomycetin (a PP1 inhibitor) did not block the high-K^+^-induced NCC dephosphorylation, tacrolimus noticeably inhibited the rapid K^+^-induced NCC dephosphorylation. We also investigated calmodulin (CM), an upstream regulator of CaN, to confirm the involvement of CaN in K^+^-induced NCC dephosphorylation. W7 (a CM inhibitor) treatment also inhibited K^+^-induced NCC dephosphorylation. Both tacrolimus and W7 treatment did not alter the expression of WNK4 and SPAK. Furthermore, oral K^+^-load-induced kaliuresis was significantly blunted in tacrolimus-treated mice. These data suggested that high K^+^ activated the CM–CaN pathway, dephosphorylating NCC and causing kaliuresis. Another group using mice lacking FKBP12 reported that there was no significant difference in WNK4, SPAK, and OSR1 expression following tacrolimus treatment [40]. They showed that BaCl_2_-induced depolarization caused NCC dephosphorylation, even with constitutive, active SPAK expression in cultured cells, and NCC dephosphorylation was clearly inhibited by tacrolimus. These data strongly support CaN as a potent phosphatase that dephosphorylates NCC under acute K^+^ conditions via a mechanism independent of the Cl^‒^–WNK4–SPAK cascade.

CaN is a calcium (Ca^2+^)- and CM-dependent serine/threonine PP. The activation of CaN requires an increase in intracellular Ca^2+^ concentration ([Ca^2+^]_in_). Therefore, we hypothesized that elevated [K^+^]_ex_ increases [Ca^2+^]_in_ to activate CaN for rapid K^+^ excretion by the kidney. Using Fluo-4 AM, we discovered that high K^+^ increases [Ca^2+^]_in_ in cultured cells (unpublished data). Further investigation is required to confirm the hypothesis. The acute K^+^ loading-induced NCC dephosphorylation mechanism is shown in Fig. 2b.

## Conclusions and implications

In this review article, we summarized that (1) K^+^ intake has beneficial effects against hypertension, CVD, and mortality; (2) NCC is a key molecule for K^+^-related BP control; and (3) NCC phosphorylation is regulated by different molecular mechanisms between the low- and high-K^+^ condition. CaN inhibitors, which are used as immunosuppressive therapy, exert side effects of hypertension and hyperkalemia. According to our findings, NCC phosphorylation might be increased in patients treated with a CaN inhibitor, even after high-K^+^ intake. Moreover, NCC is phosphorylated and activated in patients with salt-sensitive hypertension with low-K^+^ intake. Therefore, thiazide diuretics are supposed to be effective antihypertensive drugs for hypertension caused by low-K^+^ intake and CaN inhibitors.

## Electronic supplementary material

Below is the link to the electronic supplementary material.

Supplementary Material 1 (PDF 94 kb)
