# Supplementary material for: Clinical importance of potassium intake and molecular mechanism of potassium regulation
Source: Clin Exp Nephrol. 2019 Jul 17;23(10):1175–80. doi: 10.1007/s10157-019-01766-x (PMC6746677; doi:10.1007/s10157-019-01766-x)
Supplement: Supplementary file 1 — Supplementary Material 1 (PDF 94 kb) [file 10157_2019_1766_MOESM1_ESM.pdf]

Online Resource 1. List of previous rodent studies related to potassium load and WNK-SPAK-NCC cascade

	rodent strains	K administration	anion	duration	NCC	SPAK	WNK4
**van der Lubbe** [1]	**Sprague-Dawley rat**	**5% K diet /<br>low Na + high K diet**	**n.d.**	**8 days**	**tNCC ↓<br>pNCC ↓ (n.s.)**	**pSPAK ↑**	**WNK4<br>(protein) ↑**
**Rengarajan** [2]	**Sprague-Dawley rat**	**2% K diet /<br>150 mM K i.v.**	**Cl^-**	**3 h*1**	**pNCC ↓**	**pSPAK ↓ (n.s.)<br>SPAK-FL ↓ (n.s.)**	**n.d.**
**Castañeda-Bueno** [3]	**C57BL/6 mouse**	**5% K diet**	**citrate**	**4 days**	**pNCC ↑**	**pSPAK →<br>pSPAK-KS ↑**	**n.d.**
**Vitzthum** [4]	**C57BL/6 mouse**	**5% K diet**	**citrate**	**10 days**	**mRNA →<br>pNCC ↑ （n.s.)**	**mRNA →<br>protein ↑ (n.s.)**	**mRNA →<br>protein →**
**Shoda** [5]	**C57BL/6 mouse**	**1.7% K oral gavage**	**Cl^-<br>citrate<br>gluconate**	**15 min**	**pNCC ↓**	**pSPAK →**	**protein →**

*1, after overnight fasting free access to a high K diet. n.d.: no data; n.s.: not significant; i.v.: intravenous; pNCC: phosphorylated NCC; pSPAK: phosphorylated SPAK; SPAK-FL: SPAK-full length; pSPAK-KS: phosphorylated SPAK-kidney specific; tNCC: toral NCC; K^+: potassium; Cl^-: chloride.

References

[1] N. van der Lubbe *et al.*, "K+-induced natriuresis is preserved during Na+ depletion and accompanied by inhibition of the Na+-Cl- cotransporter.," *Am. J. Physiol. Renal Physiol.*, vol. 305, no. 8, pp. F1177-88, 2013.

[2] S. Rengarajan, D. H. Lee, Y. T. Oh, E. Delpire, J. H. Youn, and A. a McDonough, "Increasing plasma [K+] by intravenous potassium infusion reduces NCC phosphorylation and drives kaliuresis and natriuresis.," *Am. J. Physiol. Renal Physiol.*, vol. 306, no. 9, pp. F1059-68, 2014.

[3] M. Castañeda-Bueno *et al.*, "Modulation of NCC activity by low and high K(+) intake: insights into the signaling pathways involved.," *Am. J. Physiol. Renal Physiol.*, vol. 306, no. 12, pp. F1507-19, Jun. 2014.

[4] H. Vitzthum, A. Seniuk, L. H. Schulte, M. L. Müller, H. Hetz, and H. Ehmke, “Functional coupling of renal K+ and Na+ handling causes high blood pressure in Na+ replete mice.,” *J. Physiol.*, vol. 592, pp. 1139–57, 2014.

[5] W. Shoda *et al.*, “Calcineurin inhibitors block sodium-chloride cotransporter dephosphorylation in response to high potassium intake,” *Kidney Int.*, vol. 91, no. 2, pp. 402–411, Feb. 2017.
